# “What medical students with better academic results do: a cross-sectional analysis”

**DOI:** 10.1186/s12909-023-03999-7

**Published:** 2023-01-11

**Authors:** Amaia Urrizola, Raúl Santiago, Alfredo Gea, Sandra Rubio, Anna Vilalta-Lacarra, Javier Rodríguez, Leire Arbea

**Affiliations:** 1 Department of Medical Oncology, Clínica Universidad de Navarra, C/ Pio XII 36, 31008 Pamplona, Navarra Spain; 2 Department of Medical Education, University of Navarre, C/Irunlarrea 1, 31008 Pamplona, Navarra Spain; 3 Department of Educational Sciences, University of La Rioja, C/Luis de Ulloa Nº 2, 26004 Logroño, Spain; 4 Department of Preventive Medicine and Public Health, University of Navarre, C/Irunlarrea 1, 31008 Pamplona, Navarra Spain; 5 Department of Radiation Oncology, Clínica Universidad de Navarra, C/ Pio XII 36, 31008 Pamplona, Navarra Spain

**Keywords:** “Medical education”, “Undergraduate education”, “Academic performance”, “Learning Approach”, “Student Engagement”, “Motivation”, “Intrinsic Value”, “Self-regulation”

## Abstract

**Background:**

With university material doubling over time, medical students need to learn how to become successful life-long learners. Overall a Deep Approach (DA) to learning, and Self-Regulation (SR) skills are among the elements with a potential to accelerate learning, and Student Engagement (SE) has been associated with better university outcomes. However, specific recommendations concerning what students should do are lacking. The aim of this study was to identify above-average students’ specific attitudes and strategies toward learning.

**Methods:**

A cross-sectional analysis of the answers to the validated questionnaires Revised Study Process Questionnaire (R-SPQ-2F), SE, and Motivated Strategies for Learning Questionnaire (MSLQ) of 155 s and third-year students included in a prospective interventional study in the University of Navarre in September 2020 was performed. Students were stratified according to their standardized average mean in above-average (mean > 0) and below-average (mean ≤ 0).

**Results:**

Overall, 67.1% of students scored higher in DA than in Surface Approach (SA) and had very high Intrinsic Value (IV, median 5.9). A higher proportion of above-average students had DA > SA score (72.7% vs 57.1%, *p* = 0.05), and showed higher scores in SR (median 4.9 vs 4.3, *p* = 0.007) compared to below-average, while the latter scored higher in SA (median 24.5 vs 23, *p* = 0.04), and surface motive (median 11 vs 9, *p* = 0.007). No differences were found in SE, and both groups had average scores in the cooperative dimension. Differences were rooted to hard work, interest over material and prioritizing understanding over rote-learning motives and aligned strategies.

**Conclusions:**

Curricula design and assessment should be aligned to promote DA and SR skills among learners. Furthermore, it is paramount that teachers help instill students with interest over material and encourage understanding and hard work, since are traits associated with better results. More studies concerning metacognition and other promising traits for becoming life-long learners and prepared professionals should be made.

**Supplementary Information:**

The online version contains supplementary material available at 10.1186/s12909-023-03999-7.

## Background

The concern underlying the ability to recall knowledge learned during medical school and the ability to apply that knowledge in the clinical setting in the following years has been discussed since the nineteenth century [1]. Since on many occasions the assessment is focused on content reproduction, students tend to opt for rote learning, which can render them unable to perform appropriately in the clinical setting and deliver the expected outcomes [2]. Also, the amount of material taught during University is exponentially increasing, doubling every 10–20 years [2], and some of the content would be obsolete by the time they reach the clinical work, so it is adamant that students learn how to become “successful” life-long learners [3].

There are different tools to measure students’ attitudes and behaviors towards learning. The Learning Approach (LA) was established by Marton et al. [4] and it refers to how students tackle the learning of a specific task based on their motivations (the motive dimension) and processes (the strategy dimension). It results from the interaction of the student characteristics, teaching characteristics, and curriculum [4]. It is not a fixed trait and varies depending on the educational context [5], and particularly the assessment method. It has been argued that a Deep Approach (DA) to learning is associated with an interest to understand the material and integrate it with prior experiences, being as such regarded as “the most desirable and successful” learning approach [4]. Self-regulation (SR) describes students that are “metacognitively, motivationally, and behaviorally active in one’s learning and performance” [6], relying on their capacity to discern what they know from what they don’t. Furthermore, Student Engagement (SE) measures the adherence in terms of time and effort of students to activities linked to desirable outcomes in college [7].

When assessed in relation to learning and academic outcomes, previous data supports that a deep motivation and approach to learning, particularly the intrinsic goal orientation or Intrinsic Value (IV) and SR, are among some of the student-dependent elements with potential to accelerate learning [8]. Also, a DA to learning has been associated with better academic results [9–11]. However, there is scarce and conflicting data between different countries, and very little representation of European students concerning SR among medical students [12–14].

Furthermore, higher levels of SE have been linked to higher courses, high Problem Based Learning (PBL) presence in the curricula [15], and also better academic results [16]. Nevertheless, there are no available studies concerning the SE assessment in medical students.

Finally, these studies do not result in specific recommendations concerning what medical students should do to achieve better academic results.

On this background, this cross-sectional, observational study aims to assess, identify and highlight the specific attitudes and behaviors that characterizes students with above-average results, in contrast to the below-average, in order to guide future recommendations.

## Methods

From September 2020 to September 2021 159 pre-clinical undergraduate second and third-year medical students were recruited at the University of Navarre for an intervention study designed to assess the impact of a learning intervention in the LA, SE, IV and SR of medical students. The present observational study is a pre-planned cross-sectional analysis and presents data related to the baseline data collected on LA, SE, IV and SR in relationship to the student’s academic results in order to identify the characteristics of above-average students in contrast to the below-average.

### Student sample

Inclusion criteria were undergraduate preclinical medical students ≥ 18 years, enrolled in the second and third years at the University of Navarre. Second-year students had passed their first year of Medicine the prior academic year and began their second-year examinations during the period 2020–2021. Third-year students passed all the examinations from the second year of Medicine the prior academic year and began the third-year examinations during the 2020–2021 period. First-year students were excluded from the analysis since they were the first course in the Medicine School degree to begin with an integrated curriculum.

After a brief presentation (15 minutes) of the study characteristics before the beginning of a compulsory lecture, an invitation to participate was sent via e-mail to all the second, and third-year students. The participation was based on a voluntary basis, and all the participants that provided informed consent to participate in this study were recruited.

### Study instruments

After a literature review, out of the available validated scales the ones selected for the study based on their easy application, topic of assessment, and usefulness were Biggs et al. Revised Study Process Questionnaire (R-SPQ-2F) [5] for LA. The Deep Approach (DA) and Surface Approach (SA) dimensions were scored following the guidelines of the original paper [5]. The Afhlfeld et at. Student Engagement [15], and the Pintrich & De Groot Self-regulation (SR) and Intrinsic Value (IV) dimensions of the Motivated Strategies to Learning (MSLQ) [17] were used to assess SE, SR and IV respectively.

**Fig. 1:**
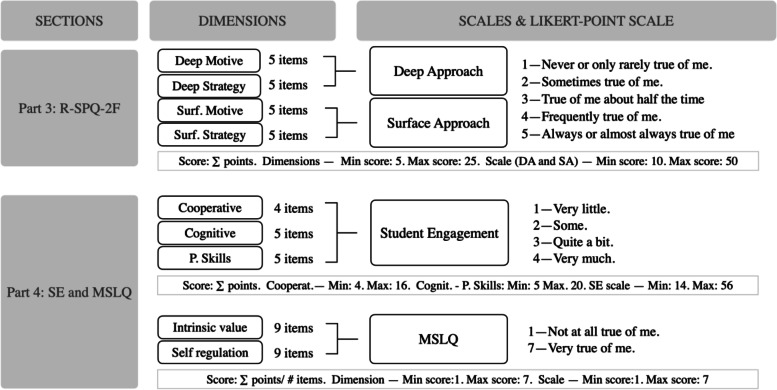
Dimensions and scoring guidelines of the employed scales. Cognit: Cognitive. Cooperat: Cooperative. DA: Deep Approach. Motivated Strategies for Learning Questionnaire. P. Skills: Personal Skills. R-SQP-2F: Revised Study Process Questionnaire. SA: Surface Approach. SE: Student Engagement

These scales were included as Part 3 and Part 4 in a multi-section questionnaire (Fig. 1) developed with Google Forms© that included items to assess demographic data (Part 1) and learning techniques (Part 2). The resulting questionnaire was sent to the students’ academic e-mail and had to answer it within a week. It was compulsory to answer all the questions in each section to continue the questionnaire.

For Study purposes, “this course” was substituted by “the courses of the Degree in Medicine” in the heading of cognitive and personal skills dimensions of the SE Questionnaire, “this class” by “the courses of medicine” in mslq13 and mslq15 of the MSLQ questionnaire, “this subject” to “the subjects” in mslq18. 

### Instruments’ reliability

Based on the data of the respective original publications of the validate scales the Cronbach’s Alpha score for the individual components of R-SPQ-2F was 0.73 for DA and 0.64 for SA (Deep Motive—DM: 0.62, Deep Strategy—DS 0.63, Surface Motive -SM 0.72, Surface Strategy—SS 0.57) [5]. For SE the alpha reliability was 0.84 for the 14-item instrument [17]. Finally, according to the MSLQ scale, the IV dimension had a Cronbach’s Alpha value of 0.87 and the SR of 0.74 [15].

Since the questionnaires were translated from the originals to Spanish, a Confirmatory Factor Analysis (CFA) was performed using Structural Equation Modeling (SEM) on a polychoric correlation matrix, and *maximum likelihood* estimation method. The Tucker-Lewis Index (TLI) and the Comparative Fit Index (CFI) were chosen as measures of incremental fit (values above 0.90 are indicative of a good fit), and the Root-Mean- Square Error of Approximation (RMSEA) was selected as a measure of parsimonious fit (values equal or below 0.05 imply a good fit to the model).

### Data collection

After providing consent, the students responded to the questionnaire sent to their academic e-mail with a time-limit to its completion of one week.

Academic results of all the students of the second and third-year of Medicine School were retrieved upon authorization from Faculty. The weighted mean from the academic year 2019–2020 constituted the baseline results. The Standardized Mean (SM) was calculated for each participant in comparison to their academic year’s mean as indicated below. Students were stratified as above-average (SM > 0), and below-average (SM ≤ 0).

$$SAM = \frac{student\mathrm{^\prime}s \ mean \ \left(over \ 10\right) -Academic \ year \ mean \ (over \ 10)}{Academic \ year \ standard \ deviation}$$

### Statistical analysis

The data collected were tabulated and analyzed using the Software for Statistics and Data Science (STATA) version 14.1 (Stata Corporation LP; College Station, TX, USA). Means with standard deviation (SD) or medians with interquartile range (IQR), and frequencies with percentages were used to present continuous and categorical variables, respectively. Chi-square test, or Fisher corrections, if necessary, were used to analyze categorical variables. Student’s T-test and Analysis of Variance (ANOVA), or Mann–Whitney U and Kruskal–Wallis where appropriate according to the distribution, were used for quantitative variables. The significance level was two-sided at *P* < 0.05.

## Results

**Fig. 2:**
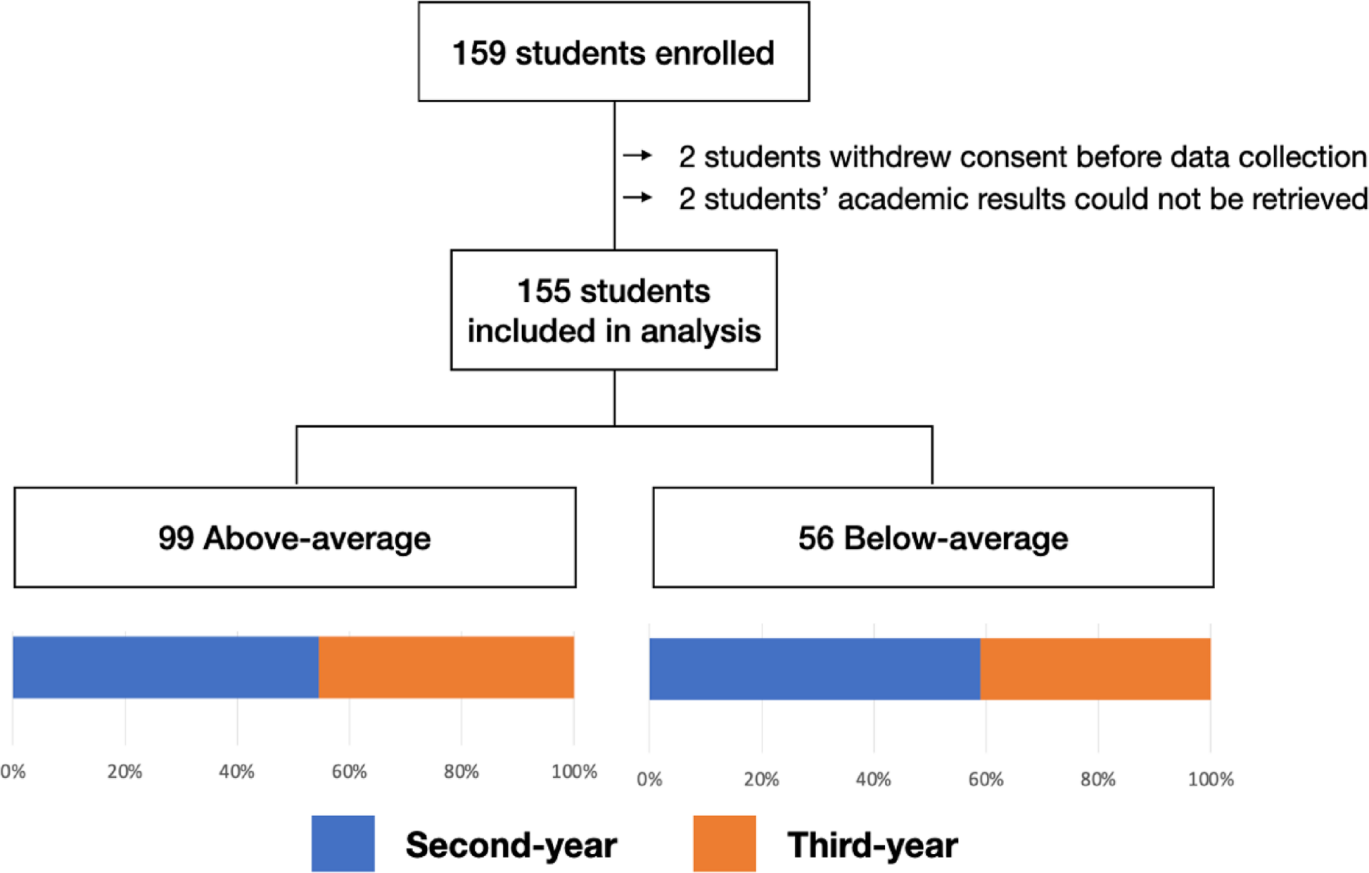
Flowchart of students included in the study, stratified by academic results

The total number of 159 students consented to participate in the study and submitted their responses to the questionnaire. Complete data were available in 155 students (96.9%): eighty-seven (56.1%) were second-year and sixty-eight (43.9%) were third-year students, which represented 34.3% of the overall university population of those years (Fig. 2). The global standardized mean was 0.31 (SD 0.8). There were 99 (63.9%) above-average (standardized mean > 0) and 56 (36.1%) below-average students (standardized academic mean ≤ 0).

There were no differences in the percentage of above-average students between courses or sex.

Overall, seventy percent were women, and the most common nationality was Spanish (96.1%). Up to 27.1% had undergone either a study technique or time management course, and less than 2% had prior grade studies. There were no statistically significant differences by academic results groups (Table 1), nor by academic year (Supplementary Table 1).

**Table 1:** Baseline demographic and academic characteristics of participants, stratified by academic results

Baseline characteristics	Global (155)	Above average (99)	Below average (56)	*p* Value
Age, mean (Sd)	19.2 (0.8)	19.2 (0.8)	19.2 (0.8)	0.88^a^
Gender, n (%)				
Women	110 (71.0)	69 (69.7)	41(73.2)	0.64^b^
Men	45 (29.0)	30 (30.3)	15 (26.8)	
Academic year, n (%)				
Second-year	87 (56.1)	54 (54.6)	33 (58.9)	0.60^b^
Third year	68 (43.9)	45 (45.5)	23 (41.1)	
Standardized mean, mean (Sd)	0.3 (0.8)	0.8 (0.1)	-0.5 (0.1)	< 0.001^a^
Spanish students, n (%)	149 (96.1)	97 (98.0)	51 (91.1)	0.06^c^
International students, n (%)	6 (3.9)	2 (2.0)	4 (7.1)	
Prior courses, n (%)	30 (19.4)	19 (19.2)	11 (19.6)	0.95^c^
Other grade studies, n (%)	3 (1.9)	2 (2.0)	1 (1.8)	0.70^c^

^a^ Mann–Whitney U

^b^ Chi-Squared Test

^c^ Fisher’s exact Test

To test whether the factor structure proposed by the original questionnaires was suitable for our data, we performed a CFA. After freeing the covariances suggested by modification indices, the modified models freeing these paths was found to have a significantly better fit (Supplementary Table 2).

There was a strong positive correlation between the DA from the R-SPQ-2F, the IV (0.6), and SR dimensions (0.6) from the MSLQ, as well as a strong negative correlation between DA and SA (-0.5). IV and SR were also strongly correlated (0.5), as were intrinsic value and personal skills dimension from the SE (0.6) (Table 2).

**Table 2:** Correlation between the dimensions of the R-SPQ-2F, Student Engagement, and MSLQ questionnaires

	**DA**	**SA**	**IV**	**SR**	**Cooperative**	**Cognitive**	**P. Skills**
DA	1.0						
SA	-0.50	1.0					
IV	0.61	-0.48	1.0				
SR	0.60	-0.32	0.54	1.0			
Cooperative	0.38	-0.16	0.21	0.27	1.0		
Cognitive	0.44	-0.35	0.40	0.41	0.16	1.0	
P. Skills	0.47	-0.28	0.56	0.45	0.35	0.46	1.0

*DA* Deep approach, *SA* Surface approach, *IV* Intrinsic Value, *SR* Self-regulation, *P. Skills* Personal skills

*R-SPQ-2F* Revised student process questionnaire, *MSLQ* Motivated strategies for learning questionnaire

Dimensions: R-SPQ-2F = Deep and surface approach. Student engagement = Cooperative, Cognitive, and Personal Skills. MSLQ = Intrinsic Value and Self-Regulation

The global results of the questionnaires with their respective Cronbach’s alfa were as described in Table 3. Overall DA score was higher than the SA, with similar values between surface and deep strategies. The cooperative values were lower than the cognitive and personal skills dimensions of the SE questionnaire, and students showed very high IV score, and high SR score. Above-average students had statistically significant higher SR score (median 4.9 vs 4.3, *p* = 0.007), while below-average had higher SA (median 24.5 vs 23, *p* = 0.04), particularly SM (median 11 vs 9, *p* = 0.007), with no differences for the other dimensions of the questionnaires. Also, more above-average students scored higher in DA than SA (DA > SA 72.7% vs 57.1%, *p* = 0.05).

**Table 3:** Learning approach, Student Engagement and Motivated Strategies for Learning Questionnaires results of 155 students, results stratified by academic results

Questionnaire	Total (*n* = 155)					Above average (*n* = 99)				Below average (*n* = 56)			*p* Value^a^
	**Mean (Sd)**	**Median**		**IQR**	**Cronbach’s alfa**	**Mean (Sd)**	**Median**		**IQR**	**Mean (Sd)**	**Median**	**IQR**	
**Deep approach**	30.0 (6.3)	30.0		26.0 – 34.0	0.82	30.4 (5.9)	31.0		27.0 -34.0	29.2 (7.1)	29.5	23.5 – 34.0	0.28
Deep motive	15.9 (3.4)	16.0		14.0 – 18.0	0.66	16.2 (3.2)	17.0		15.0 – 18.0	15.3 (3.7)	15.0	12.5 – 18.0	0.14
Deep strategy	14.1 (3.5)	14.0		11.0 – 16.0	0.69	14.2 (3.2)	14.0		12.0 – 16.0	13.8 (3.9)	14.0	10.5 – 16.5	0.53
**Surface approach**	24.5 (6.6)	24.0		20.0 – 29.0	0.81	23.8 (6.9)	23.0		19.0 – 28.0	25.8 (5.8)	24.5	21.0 – 29.5	0.04
Surf. motive	10.5 (3.4)	10.0		8.0 – 13.0	0.66	10.1 (3.5)	9.0		8.0 – 12.0	11.3 (3.0)	11.0	9.0 – 13.5	0.007
Surf. strategy	14.0 (3.8)	14.0		11.0 – 16.0	0.7	13.7 (4.0)	13.0		11.0 – 17.0	14.5 (3.5)	14.0	12.0 – 16.0	0.25
**DA > SA, n (%)**	104 (67.1)				-	72 (72.7)				32 (57.1)			0.05
**SA ≥ DA, n (%)**	51 (32.9)				-	27 (27.3				24 (42.9)			
**SE**	37.4 (5.8)		37.0	34.0 – 42.0	0.76	37.6 (5.2)		37.0	35.0 – 41.0	36.9 (6.8)	35.5	31.5 – 42.0	0.39
Cooperative	9.0 (2.5)		9.0	7.0 – 10.0	0.66	8.9 (2.3)		8.0	7.0 – 10.0	9.0 (2.7)	9.0	7.0 – 11.5	0.68
Cognitive	13.7 (2.5)		14.0	12.0 – 15.0	0.58	13.9 (2.6)		14.0	12.0 – 16.0	13.4 (2.3)	13.0	12.0 – 15.0	0.31
Pers. Skills	14.7 (2.9)		15.0	13.0 – 17.0	0.64	14.9 (2.6)		15.0	14.0 – 17.0	14.5 (3.5)	15.0	11.0 – 18.0	0.67
**MSLQ**													
Intrinsic Value	5.8 (0.8)		5.9	5.3 – 6.3	0.85	5.9 (0.7)		6.0	5.4 – 6.3	5.6 (0.9)	5.6	5.1 – 6.3	0.13
Self-Regulation	4.7 (0.8)		4.8	4.2 – 5.2	0.69	4.8 (0.7)		4.9	4.4 – 5.2	4.4 (1.0)	4.3	3.8 – 5.1	0.007

*DA* Deep approach, *SA* Surface approach, *IQR* Interquartile range, *MSLQ* Motivated strategies to learning questionnaire

^a^ Mann–Whitney U Test

A comparative analysis per individual item was performed to ascertain the root of the differences (Table 4). There were 13 statements in which statistically significant differences were found between both groups. Seven were motivations and processes about understanding the material, for which above average showed higher agreement for making sure they understood the material (by testing and asking questions: mslq21, ds10), considering this important (mslq18), and trying to learn from mistakes even despite getting poor results in tests (mslq12). In contrast, below-average showed higher rates of agreement with rote memorization (aiming for a reproduction of the content, even compromising understanding: sm11, ss8, se5). Four of the statements were about hard work and commitment, for which above-average had higher rates for continue working over dull and uninteresting material (mslq29) or working hard to achieve good grades despite disliking the content (mslq39), while the below-average scored higher in giving up or focusing on the easy parts when work was hard (mslq23), or keeping the work to the minimum since they did not find the course interesting (sm7).

**Table 4:** Statements with significant differences in scores, stratified by academic performance

**Question**		Above Average		Below Average		*p* Value^a^
		Mean	p50	Mean	p50	
**Revised-Students Processes Questionnaire-2 Factor**						
**DM**	1. I find that at times studying gives me a feeling of deep personal satisfaction	3.8	4	3.4	3	0.008
**DS**	10. I test myself on important topics until I understand them completely	3.6	4	3.0	3	0.002
**SM**	7. I do not find my course very interesting, so I keep my work to the minimum	1.6	1	1.9	2	0.05
	11. I find I can get by in most assessments by memorizing key sections rather than trying to understand them	2.1	2	2.5	2	0.007
** SS**	8. I learn some things by rote. Going over and over them until I know them by heart even if I do not understand them	2.6	3	3.1	3	0.04
1: Never or only rarely true of me., 2: Sometimes true of me, 3: True of me about half the time, 4: Frequently true of me, 5: Always or almost always true of me						
**Student Engagement**						
**Cooperative**	2. Worked with other students on projects during class time	2.0	2	2.4	2	0.01
**Cognitive**	5. Memorizing facts. Ideas or methods from your course and readings so you can repeat them in almost the same form	2.5	3	2.8	3	0.02
1 — Very little. 2 — Some. 3 — Quite a bit. 4 — Very much						
**Motivated Strategies for Learning Questionnaire**						
**IV**	12. Even when I do poorly on a test, I try to learn from my mistakes	6.0	6	5.5	6	0.02
	18. Understanding this subject is important to me	6.6	7	6.1	6	< 0.001
**SR**	21. I ask myself questions to make sure I know the material I have been studying	5.3	6	4.3	5	< 0.001
	23. When work is hard I either give up or study only the easy parts. (*R)	2.5	2	3.1	3	0.04
	29. Even when study materials are dull and uninteresting. I keep working until I finish	5.6	6	4.9	5	0.006
	39. I work hard to get a good grade even when I don’t like a class	6.1	6	5.6	6	0.01
1 – Not at all true to me 7 – Very true of me						

*DM* Deep motive, *DS* Deep strategy, *SM* Surface motive, *SS* Surface strategy, *IV* Intrinsic value, *SR* Self-regulation

^a^ Mann–Whitney U Test

Finally, nearly 75% of above-average students reported that “feeling a deep personal satisfaction at times while they were studying” (dm1) was frequently or always true for them, while 50% of below-average reported it was sometimes or half of the time true for them. Interestingly, overall students reported low agreement with working with others on projects during class time (se2), but the above average were less prone to it.

## Discussion

Our data show that above-average students had higher SR score while below-average presented higher SA, particularly SM, with no differences for the other dimensions of the questionnaires.

Intrinsic motivation affirmations such as a feeling of deep personal satisfaction through their study, a deep interest in understanding the content, and learning from mistakes, and strategies that were aligned with these motivations were more frequent in above-average students. In stark contrast, those below average didn’t find the content interesting, which led them to not being motivated to hard-work and prioritized rote learning.

Consistent with other measures in medical students [18], overall, DA score was significantly higher than the SA. It is important to bear in mind, though, that the differences were linked to the motivation, with students showing higher DM values than SM, while there were no differences in the overall process score (DS and SS scores), which may be linked to an aim on information reproduction linked to multiple choice question assessment of the university [19].

The learning approach reflects how students tackle a specific learning task [20]. An intrinsic motivation and interest for understanding and integrating the to-be learnt material with previous experiences and knowledge is what characterizes a DA to learning. As such, the DA has been considered the desirable LA for meaningful learning [4].

In Hattie’s review a deep motivation traits are deemed to have potential to impact learning, while surface motivation and approach were likely to have a negative impact [8, 21].

When considering the academic results, previous studies performed in medical students show that those with better academic score [9–11]. In our sample, both above and below average students showed high DA score. Nevertheless, a higher proportion of above-average presented a DA > SA score. Concerning the absolute score, however, the main differences in the approach to learning were linked to the SA, instead of the DA, with below-average showed significantly higher SA, particularly SM.

Student engagement measures cognitive, behavioral, and emotional elements that impact learning outcomes [7]. Higher SE levels are linked to higher academic courses [15] and better academic results [16]. Although there is no prior available data for SE in medical students, students in our cohort present SE score similar to the results obtained in other degrees, with students showing high scores for cognitive and personal skills dimensions[15].

Problem Based Learning (PBL) represents one of the strategies developed in the paradigm-shift from passive students to actively-engaged students in order to enable students to become life-long learners [15], and is associated with higher SE levels. Interestingly, despite devoting an increasing percentage of the curricula to team-based learning and problem-based learning methodologies, still the cooperative scores are significantly low in our sample. Moreover, there were no significant differences between above and below-average students concerning the SE.

Additionally, students showing high levels of SR are able to appropriate allocate the use of resources to reach their learning objectives, which is paramount for self-directed and life-long learning [22, 23]. Intrinsic Value (IV) and SR, are among some of the student-dependent elements with potential to accelerate learning [8]. Consistent with previous data, our population presented very high IV score and high SR score [12, 13]. Although the relationship with academic results shows conflicting results [12–14], in our cohort above-average students had statistically significant higher SR score.

To our knowledge, this is the first study to perform a multidimension assessment in a European sample of medical students considering the learning approach, student engagement and motivation, particularly intrinsic value, and self-regulation, and relating them with the academic results. The Cronbach’s alfas of our sample are comparable to those of the original studies, ensuring internal consistency. As expected, our data show a strong positive correlation between the DA of the R-SPQ-2F, the IV, and SR dimensions of the MSLQ questionnaire, and a strong negative correlation with the SA of the R-SPQ-2F.

With the aim of getting some insight regarding what attitudes and strategies medical students with better academic results have, in order to guide future recommendations for undergraduate students, we performed a comparative analysis per individual item. Our data show that above-average students are characterized by feeling a deep personal satisfaction through their study, and a deep interest in understanding the content, and learning from mistakes, aligning their strategies with these motivations, whereas, in stark contrast, those below average didn’t find interesting the topic, thus were not motivated to hard-work, and prioritized rote learning.

Another strength of our data is that the academic results of all the students in both courses (included and not included in the study) were available which allowed us to identify the traits of the "real" above-average students, and not only those with better results in our cohort. Since there were no significant differences in the distribution of above and below-average students per course, it is safe to assume the differences were based on individual traits, and not in course differences.

There are, however, some limitations to bear in mind. Firstly, the participants in our sample were recruited in a voluntary basis, so probably the inherently motivated students were the ones to sign-up. This may explain the high DA, cognitive and personal skills, IV, and SR values of our cohort, and may account to some of the discrepancies respective to prior data. Furthermore, students were from preclinical courses, so our results may not be representative of all the medical student’s population. Additionally, it could have limited our ability to find greater differences in students’ attitudes.

It should be taken into account that the academic results pertained to the prior course and were used as proxies for the baseline academic results of each of the participants. Since no known intervention occurred during the summer, and there were no differences for the prior courses receive between above and below-average students, it is safe to assume that there would be no significant differences.

Another limitation is that we highlight desirable traits under the assumption that good professional performance is associated to good academic results. Nevertheless, we “wrongly” assume that the content assessed with the university tests guarantee students will be able to retain this information in the long-term [24] and apply it. Medical graduates report feeling unprepared to face some of the challenges when they start working in the hospital, and their degree of confidence and preparedness is usually higher than those referred by the stakeholders or supervisors [25, 26]. Also, given the speed of new medical advancements, it is paramount that students develop the necessary habits for self-directed learning and life-long learning, since much of what is learnt may become obsolete in the coming years. That is why identifying how to best convey and instill these skills during medical school should be addressed.

Finally, our results are limited to the items assessed in the questionnaires. There are other elements in Hattie’s review such as self-efficacy, self-judgement and reflection meta-cognition strategies that rank higher in their potential to considerable accelerate learning review [8, 21], with promising results in medical students [23, 27, 28] and that should be the focus of future learning interventions.

## Conclusions

Successful medical students score higher in DA than SA, and show high SR. Curricula design and assessment should be aligned to promote DA and SR skills among learners. Above-average students find a feeling of deep personal satisfaction through their study, and show deep interest in understanding the content, and learning from mistakes, aligning their strategies with these motivations, whereas those below-average prioritize rote learning and are not motivated to hard-work. It is paramount that teachers and tutors help instill students with interest over material and encourage understanding, perseverance, and hard work, since are traits associated with better results. More studies should be made in this regard, and in identifying traits for becoming successful life-long learners and prepared professionals.

## Supplementary Information

**Additional file 1**: **Supplementary Table 1.** Baseline demographic and academic characteristics of participants, stratified by academic year. **Supplementary Table 2.** Goodness-of-fit indices for each adjusted questionnaire for the total sample (155).

## Data Availability

The datasets used and/or analysed during the current study are available from the corresponding author on reasonable request.

## Abbreviations

ANOVA: Analysis of variance
CFA: Confirmatory factor analysis
CFI: Comparative fit index
Cognit: Cognitive
Cooperat: Cooperative
DA: Deep approach
DM: Deep motive
DS: Deep strategy
IQR: Interquartile range
IV: Intrinsic value
LA: Learning approach
Max: Maximum
Min: Minimum
MSLQ: Motivated strategies for learning questionnaire
P. Skills: Personal skills
PBL: Problem based learning
R-SPQ-2F: Revised study process questionnaire
RMSEA: Root-mean- square error of approximation
SA: Surface approach
SAM: Standardized academic mean
SD: Standard deviation
SE: Student engagement
SEM: Structural equation modeling
SM: Surface motive
SR: Self-regulation
SS: Surface strategy
STATA: Software for statistics and data science
TLI: Tucker-Lewis index
