# Supplementary material for: “What medical students with better academic results do: a cross-sectional analysis”
Source: BMC Med Educ. 2023 Jan 11;23:19. doi: 10.1186/s12909-023-03999-7 (PMC9835338; doi:10.1186/s12909-023-03999-7)
Supplement: Supplementary file 1 — Additional file 1: Supplementary Table 1. Baseline demographic and academic characteristics of participants, stratified by academic year. Supplementary Table 2. Goodness-of-fit indices for each adjusted questionnaire for the total sample (155). [file 12909_2023_3999_MOESM1_ESM.docx]

**Supplementary Table 1**. Baseline demographic and academic characteristics of participants, stratified by academic year

| **Baseline characteristics** | **Global n = 155** |  | **Second Year n = 87** |  | **Third Year n = 68** |  | **p Value** | |
| --- | --- | --- | --- | --- | --- | --- | --- | --- |
| **Age, mean (sd)** | 19.2 (0.8) |  | 18.7 (0.6) |  | 19.8 (0.6) |  | <0.001^a^ | |
| **Gender, n (%)** |  |  |  |  |  |  |  | |
| Women | 110 (71.0) |  | 64 (73.6) |  | 46 (67.7) |  | 0.45^b^ | |
| Men | 45 (29.0) |  | 23 (26.4) |  | 22 (32.4) |  |  |  |
| **Standardized mean, mean (sd)** | 0.3 (0.8) |  | 0.24 (0.7) |  | 0.39 (0.8) |  | 0.21^a^ | |
| **Academic results, n (%)** |  |  |  |  |  |  |  | |
| Above average | 99 (63.9) |  | 54 (62.1) |  | 45 (66.2) |  | 0.60^b^ | |
| Below average | 56 (36.1) |  | 33 (37.9) |  | 23 (33.8) |  |  |  |
| **Spanish students, n (%)** | 149 (96.1) |  | 82 (94.3) |  | 67 (98.5) |  | 0.89^c^ | |
| **International students, n (%)** | 6 (3.9) |  | 5 (5.8) |  | 1 (1.5) |  |  |  |
| Dutch | 1 (0.7) |  | 1 (1.2) |  | 0 |  |  |  |
| French | 1 (0.7) |  | 1 (1.2) |  | 0 |  |  |  |
| Greek | 1 (0.7) |  | 0 (0) |  | 1 (1.5) |  |  |  |
| Panamanian | 1 (0.7) |  | 1 (1.2) |  | 0 |  |  |  |
| Polish | 1 (0.7) |  | 1 (1.2) |  | 0 |  |  |  |
| Turkish | 1 (0.7) |  | 1 (1.2) |  | 0 |  |  |  |
| **Prior courses, n (%)** |  |  |  |  |  |  |  | |
| Study techniques course | 24 (15.5) |  | 15 (17.2) |  | 9 (13.2) |  | 0.48^b^ | |
| Time management course | 18 (11.6) |  | 8 (9.2) |  | 10 (14.7) |  | 0.30^b^ | |
| **Other grade studies, n (%)** | 3 (1.9) |  | 3 (3.5) |  | 0 (0) |  | 0.26^c^ | |
| ^a^ Student’s T Test ^b^ Chi-Squared Test ^c^ Fisher’s exact test. | | | | | | | |  |

**Supplementary Table 2**. Goodness-of-fit indices for each adjusted questionnaire for the total sample (155).

| **Questionnaire** | **Chi-squared** | **d.f.** | **Ratio Chi2/df** | **RMSEA** | **CFI** | **TLI** |  |
| --- | --- | --- | --- | --- | --- | --- | --- |
| **R-SPQ-2F** | 243.94^a^ | 160 | 1.52 | 0.058 | 0.96 | 0.88 |  |
| **SE** | 92.44^a^ | 73 | 1.27 | 0.04 | 0.95 | 0.94 |  |
| **MSLQ** | 185.56^a^ | 124 | 1.5 | 0.056 | 0.93 | 0.91 |  |
| d.f. = Degrees of Freedom. RMSEA = Root Mean Squared Error of Approximation. CFI = Comparative Fit Index.  TLI = Tucker-Lewis Index R-SPQ-2F = Revised Student Process Questionnaire. SE = Student Engagement.  MSLQ = Motivated Strategies for Learning Questionnaire  ^a^ p < 0.001 | | | | | | | |
